# Target Tracking with Sensor Navigation Using Coupled RSS and AoA Measurements

**DOI:** 10.3390/s17112690

**Published:** 2017-11-21

**Authors:** Slavisa Tomic, Marko Beko, Rui Dinis, João Pedro Gomes

**Affiliations:** 1 ISR/IST, LARSyS, Universidade de Lisboa, 1049-001 Lisbon, Portugal; jpg@isr.ist.utl.pt; 2 CICANT-CIC.DIGITAL, Universidade Lusófona de Humanidades e Tecnologias, Campo Grande 376, 1749-024 Lisboa, Portugal; beko.marko@ulusofona.pt; 3 CTS/UNINOVA, Campus da FCT/UNL, Monte de Caparica, 2829-516 Caparica, Portugal; 4 Instituto de Telecomunicações, Av. Rovisco Pais 1, Torre Norte, piso 10, 1049-001 Lisboa, Portugal; rdinis@fct.unl.pt; 5 Dep.^o^ de Eng.^a^ Electrotécnica, FCT/UNL, 2829-516 Caparica, Portugal

**Keywords:** target tracking, sensor navigation, received signal strength (RSS), angle of arrival (AoA), maximum a posteriori (MAP) estimator, Kalman filter (KF)

## Abstract

This work addresses the problem of tracking a signal-emitting mobile target in wireless sensor networks (WSNs) with navigated mobile sensors. The sensors are properly equipped to acquire received signal strength (RSS) and angle of arrival (AoA) measurements from the received signal, while the target transmit power is assumed not known. We start by showing how to linearize the highly non-linear measurement model. Then, by employing a Bayesian approach, we combine the linearized observation model with prior knowledge extracted from the state transition model. Based on the maximum a posteriori (MAP) principle and the Kalman filtering (KF) framework, we propose new MAP and KF algorithms, respectively. We also propose a simple and efficient mobile sensor navigation procedure, which allows us to further enhance the estimation accuracy of our algorithms with a reduced number of sensors. Model flaws, which result in imperfect knowledge about the path loss exponent (PLE) and the true mobile sensors’ locations, are taken into consideration. We have carried out an extensive simulation study, and our results confirm the superiority of the proposed algorithms, as well as the effectiveness of the proposed navigation routine.

## 1. Introduction

The problem of accurate localization of a moving object in real time has motivated a great deal of scientific research recently, owing to a constant growth of the range of enabling devices and technologies and the requirement for seamless solutions in location-based services [1,2,3,4,5,6,7,8,9,10,11,12,13,14,15,16,17,18]. In order to maintain low implementation cost, making use of existing technologies (such as terrestrial radio frequency sources) when providing a solution to the object tracking problem is strongly encouraged. Spatial information may be inferred from measurements that include, for example, time of arrival [8,10,16], received signal strength (RSS) [6,11,12], angle of arrival (AoA) or a combination of these [7,14,15,18].

The authors in [8,9,10,11,12,13,14,15,16,17,18] considered only a classical target localization problem, where they disregarded any prior knowledge and gave all importance to observations exclusively. The works in [3,4,7] investigated target tracking problems where the observations were combined with some prior knowledge to enhance the estimation accuracy. However, they all examined the purely RSS-based target tracking problem only. In [6], the authors investigated the target tracking problem by employing hybrid, RSS and AoA, measurements. The authors first linearized the highly non-linear measurement model, and on top of the linearized model, they applied a Kalman filter (KF). Therefore, the main contribution of the work in [6] is the linearization technique, since a direct application of the KF to the considered non-linear measurement model is not possible. In [6], the measurement model was linearized by using a very simple and intuitive approach. It can be summarized as forming a line and using the RSS measurements to determine the length of the line. At one point of the line, the authors situated a known anchor and used the AoA to determine the slope of the line. In that way, an estimate of the target location was obtained at the other point of the line. Although this is an effective way to tackle the non-linearity of the measurement model, the authors in [6] treated all links as equal, and no mitigation technique was used to deal with potentially negative impact from distant links. Besides the KF, a particle filter (PF) algorithm was also proposed in [6], as well as a generalized pattern search method for estimating the path loss exponent (PLE) for each link in every time step. Nevertheless, the authors considered the tracking problem with static anchors only. In addition to the mentioned algorithms specifically designed for RSS- and RSS-AoA-based target tracking, various other traditional methods (essentially modifications of the KF) are available in the literature. For instance, the extended KF (EKF) [19,20,21,22] requires no assumptions about the linearity of the state or observation models. Instead, it approximates the non-linear models by their first-order Taylor series expansion (which requires calculation of the Jacobian matrix). The unscented KF (UKF) utilizes the unscented transformation, which is founded on the intuition that it is easier to approximate a probability distribution than it is to approximate an arbitrary non-linear function or transformation [23]. The basic idea is to represent the state distribution by a minimal set of carefully selected points, called sigma points. These points completely capture the mean and covariance of the distribution, and when passed through the non-linear system, they capture the mean and covariance up to the third order (Taylor series expansion) for any non-linearity [23,24,25]. Similarly to the UKF, the PF approximates the posterior probability distribution function (PDF) of the state with sample points, called particles, but with the key difference that these particles are selected at random. Essentially, it is an ordinary randomization technique whose performance and computational complexity are directly proportional to the number of particles used [3,6]. The particles are iteratively updated according to new observations, and no linearity nor Gaussianity assumptions are required [25,26]. Moreover, the works presented in [27,28] tackled the target tracking problem with mobile sensors. Still, hybrid RSS-AoA target tracking was not a part of their study.

In this work, the problem of tracking a mobile target by employing hybrid RSS-AoA measurements is considered. We assume that the target transmit power is unknown and start by describing our linearization process of the highly non-linear observation model. The proposed linearization technique is fundamentally different from the existing one described in [6], since we apply a Cartesian to polar (spherical for three-dimensional space) conversion to deal effortlessly with the non-linear terms in the measurement model. Moreover, in contrast to [6], where the authors treat all links as equal, here we apply weights to give more importance to nearby links and mitigate the potential negative impact of remote ones. Next, we describe the target state by its location and velocity, and we incorporate the prior knowledge given by the state transition model and previous target state together with the linearized model in order to enhance the estimation accuracy. Then, by following the maximum a posteriori (MAP) criterion, we propose a novel MAP algorithm to efficiently track the target in real time. We also show that the application of the KF criterion on top of the derived linearized measurement model is straightforward, resulting in a novel KF algorithm. Finally, we propose a simple sensor navigation routine, which leads to great improvement in the estimation accuracy, even for a low number of sensors. A realistic scenario where the PLE and the true sensors’ locations are not perfectly known is also taken into consideration. Compared with the existing KF algorithm for RSS-AoA target tracking in [6], i.e., the existing linearization technique, the proposed KF algorithm lowers the estimation error by about 1 m, or roughly 25%. Furthermore, in comparison with other existing and well-known methods, such as EKF, UKF and PF, the proposed MAP algorithm matches their performance with lower execution time, and in our simulations, it always converged, independently of the chosen initialization point, in contrast to the existing methods.

This paper is organized as follows. In Section 2, we introduce the target state transition model, as well as the measurement model, and we formulate the target tracking problem in a Bayesian framework. Section 3 describes our technique to linearize the measurement model. Section 4 presents the proposed tracking algorithms, as well as the proposed sensor navigation routine for mobility management. In Section 5, simulation results are presented for two different target trajectories in order to validate the performance of the proposed algorithms. Finally, Section 6 summarizes the main conclusions.

## 2. Problem Formulation

We consider a wireless sensor network (For simplicity, and without loss of generality, this paper focuses on two-dimensional scenarios. The extension to three dimensions is straightforward.) (WSN) composed of *N* mobile sensors with known locations, $a_{i,t}={\left[a_{ix,t},a_{iy,t}\right]}^{T}$ for i=1,…,N, and a moving target whose location, $x_{t}={\left[x_{x,t},x_{y,t}\right]}^{T}$, we wish to determine at each time instant *t*. We assume an almost constant velocity target motion model (e.g., perturbed only by wind gust) such that the velocity components in the x and y directions at time *t* are given by:

(1) $$v_{t}=v_{t-1}+r_{v,t},$$

where $r_{v,t}$ represents the noise perturbations. Hence, from the equations of motion [19], the target location at time *t* is:

(2) $$x_{t}=x_{t-1}+v_{t-1}\Delta +r_{x,t},$$

where Δ and $r_{x,t}$ are the sampling interval between two consecutive time steps and location process noise, respectively. Now, if we describe the target state at *t* by its location and velocity, i.e., $\theta_{t}={\left[x_{t}^{T},v_{t}^{T}\right]}^{T}$, from (1) and (2), we get:

(3) $$\theta_{t}=S\;\theta_{t-1}+r_{t},$$

where $r_{t}={\left[r_{x,t}^{T},r_{v,t}^{T}\right]}^{T}$ is the state process noise [2,3,4,5,6,7], assumed to be zero-mean Gaussian with covariance matrix Q, i.e., $r_{t}∼N\left(0,Q\right)$. The symbol S in (3) stands for the state transition matrix. The noise covariance and the state transition matrices are given by:

$$Q=q\left[\begin{array}{cccc} \frac{\Delta^{3}}{3} & 0 & \frac{\Delta^{2}}{2} & 0\\ 0 & \frac{\Delta^{3}}{3} & 0 & \frac{\Delta^{2}}{2}\\ \frac{\Delta^{2}}{2} & 0 & \Delta & 0\\ 0 & \frac{\Delta^{2}}{2} & 0 & \Delta \end{array}\right]\;,\;\;\;S=\left[\begin{array}{cccc} 1 & 0 & \Delta & 0\\ 0 & 1 & 0 & \Delta \\ 0 & 0 & 1 & 0\\ 0 & 0 & 0 & 1 \end{array}\right]\;,$$

with *q* denoting the state process noise intensity [2,4,29].

At each time instant, the target emits a signal to sensors, which extract both the RSS and AoA information from it. Thus, the measurement equation can be formulated as:

(4) $$z_{t}=h\left(y_{t}\right)+n_{t},$$

where $z_{t}={\left[P_{t}^{T},\phi_{t}^{T}\right]}^{T}$ ($z_{t}\in R^{2N}$) is the observation vector comprising RSS, $P_{t}={\left[P_{i,t}\right]}^{T}$, and AoA, $\phi_{t}={\left[\phi_{i,t}\right]}^{T}$, measurements at time instant *t*, with $P_{i,t}$ and $\phi_{i,t}$ denoting the RSS and AoA measurement of the *i*-th anchor at time instant *t*. The function $h\left(y_{t}\right)={\left[h_{i}\left(y_{t}\right)\right]}^{T}$ in (4), where $y_{t}={\left[x_{t}^{T},\rho \right]}^{T}$ represents the vector of all unknowns (the location of the target, $x_{t}$, and $\rho =\exp\left(\frac{P_{0}}{\eta \gamma }\right)$ being a term corresponding to unknown transmit power, as we will show in Section 3) at time instant *t*, is defined as $h_{i}\left(y_{t}\right)=P_{0}-10\gamma \log_{10}\frac{∥x_{t}-a_{i,t}∥}{d_{0}}$ for i=1,…,N [30], and $h_{i}\left(x_{t}\right)=\tan^{-1}\left(\frac{x_{y,t}-a_{iy,t}}{x_{x,t}-a_{ix,t}}\right)$ for i=N+1,…,2N [31], where $P_{0}$ (dBm) is the reference power at a distance $d_{0}$ ($d_{0}=1$ m, usually) and γ is the PLE. The measurement noise, $n_{t}$, is modeled as $n_{t}∼N\left(0,C\right)$, where the noise covariance is defined as $C=\mathrm{diag}(\left[\sigma_{n_{i}}^{2},\sigma_{m_{i}}^{2}\right])$, with $\sigma_{n_{i}}$ (dB) and $\sigma_{m_{i}}$ (rad) being the noise standard deviation of the RSS and AoA measurements, respectively.

In Bayesian estimation theory, the prior knowledge, obtained through the state transition model (3), is combined with the noisy observations (4) to obtain the marginal posterior PDF, $p(\theta_{t}|z_{1:t})$. Through $p(\theta_{t}|z_{1:t})$, we can quantify the belief we have in the values of the state $\theta_{t}$ given all the past measurements $z_{1:t}$, and we can obtain an estimate at any time instant we desire. The main steps of the Bayesian estimation are described below [2,3,4,5,6,7].

- Initialization: The marginal posterior PDF at t=0 is set to the prior PDF $p(\theta_{0})$ of $\theta_{0}$.
- Prediction: By using the state transition model (3), the predictive PDF of the state at *t* is given by:
  (5) $$p\left(\theta_{t}|z_{1:t-1}\right)=\int p\left(\theta_{t}|\theta_{t-1}\right)p\left(\theta_{t-1}|z_{1:t-1}\right)d\theta_{t-1}.$$
- Update: By following Bayes’ rule [2,29], we have:

(6) $$p\left(\theta_{t}|z_{1:t}\right)=\frac{p\left(z_{t}|\theta_{t}\right)p\left(\theta_{t}|z_{1:t-1}\right)}{p(z_{t}|z_{1:t-1})},$$

where $p(z_{t}|\theta_{t})$ is the likelihood and $p\left(z_{t}|z_{1:t-1}\right)=\int p\left(z_{t}|\theta_{t}\right)p\left(\theta_{t}|z_{1:t-1}\right)d\theta_{t}$ is just a normalizing constant, independent of $\theta_{t}$, needed to ensure that $p(\theta_{t}|z_{1:t})$ integrates to one [19]. In general, the marginal PDF at t−1 cannot be calculated analytically, and the integral in (5) cannot be obtained analytically if the state model is non-linear. Therefore, some approximations are required to obtain $p(\theta_{t}|z_{1:t})$.

In the next section, we show how to efficiently linearize the non-linear measurement model in (4) for small noise power, for both the cases of known and unknown target transmit power. This technique is a crucial part of our work, since the linearized measurement model will be used as a base for the development of our algorithms in Section 4. Our linearization technique is different from the one used in the EKF, since it does not require calculation of the first-order partial derivatives. This simplifies substantially the linearization process, since calculating Jacobian matrices can be a very arduous (sometimes even not possible) and error-prone process, which might even lead to divergence of the filter [21,22,23,24].

## 3. Linearization of the Measurement Model

In this section, we neglect the prior knowledge completely and concentrate on the measurement model exclusively. We describe our linearization technique in detail, and we show that, by applying a least squares (LS) criterion to the derived model, it can be seen as an approximation of the likelihood function in (6). Thus, an estimate of the target location can be readily obtained from the derived model. However, because the prior knowledge is neglected here, we will refer to this approach as a naive one.

The considered measurement model (RSS-AoA) is highly non-linear, which makes the likelihood, i.e., the marginal PDF, non-convex. To deal with the non-convexity, one could resort to convex relaxation techniques and convert the non-convex problem into a convex one [32]. Nevertheless, this would severely raise the computational complexity (and thus, the execution time) of an algorithm, making it difficult to use it in real-time applications. Therefore, in this section, we show how to efficiently linearize the measurement model without engaging convex relaxation techniques, and we do so for both cases of known and unknown transmit power. Furthermore, by applying an LS criterion to the derived linearized measurement model, we show that, if we disregard the prior knowledge, an estimate of the target location can be obtained directly from the derived model. This estimate represents a solution to the classical localization problem (not the tracking one) and will be used for comparison in Section 5 in order to demonstrate the importance of the prior knowledge for Bayesian approaches.

In practice, network testing and calibration are often not the priority, especially in low-cost systems that use RSS as a surrogate for range [1]. Hence, some parameters, such as target transmit power, might not be calibrated, i.e., not known beforehand. Not knowing the transmit power matches not knowing $P_{0}$ in (4) [1].

From the RSS measurement model in (4), we can write:

(7) $$\exp\left(\frac{P_{0}-P_{i,t}+n_{i}}{\eta \gamma }\right)=∥x_{t}-a_{i,t}∥,\;\;\mathrm{for}\;\;i=1,\ldots ,N,$$

where we used the fact that $d_{0}=1$ m and $\eta =\frac{10}{\ln(10)}$. By breaking down the first term on the left-hand side, (7) can be rewritten as:

(8) $$\rho \;\exp\left(\frac{n_{i}}{\eta \gamma }\right)=\mu_{i,t}∥x_{t}-a_{i,t}∥,\;\;\mathrm{for}\;\;i=1,\ldots ,N,$$

where $\rho =\exp\left(\frac{P_{0}}{\eta \gamma }\right)$ and $\mu_{i,t}=\exp\left(\frac{P_{i,t}}{\eta \gamma }\right)$. Assuming that the noise term is sufficiently low, we can use the first-order Taylor series approximation (of the form exp(φ)≈1+φ for small φ), to approximate (8) as:

(9) $$\rho +\epsilon_{i,t}\approx \mu_{i,t}∥x_{t}-a_{i,t}∥,\;\;\mathrm{for}\;\;i=1,\ldots ,N,$$

where $\epsilon_{i,t}∼N\left(0,{\left(\frac{\rho }{\eta \gamma }\sigma_{n_{i}}\right)}^{2}\right)$. Then, (9) yields:

(10) $$\epsilon_{i,t}\approx \mu_{i,t}∥x_{t}-a_{i,t}∥-\rho ,\;\;\mathrm{for}\;\;i=1,\ldots ,N.$$

By converting from Cartesian to polar coordinates, we can express $x_{t}-a_{i,t}=r_{i,t}u_{i,t}:r_{i,t}\geq 0$, $∥u_{i,t}∥=1$, where the unit vector can be approximated by employing the available AoA information, i.e., $u_{i,t}={\left[\cos\left(\phi_{i,t}\right),\sin\left(\phi_{i,t}\right)\right]}^{T}$. If we apply this conversion in (10) and multiply by $u_{i,t}^{T}u_{i,t}$, we get:

(11) $$\epsilon_{i,t}\approx \mu_{i,t}u_{i,t}^{T}\left(x_{t}-a_{i,t}\right)-\rho ,\;\;\mathrm{for}\;\;i=1,\ldots ,N.$$

Similarly, for small noise power, from the AoA model in (4), we can write:

(12) $$c_{i,t}^{T}\left(x_{t}-a_{i,t}\right)\approx 0,\;\;\mathrm{for}\;\;i=N+1,\ldots ,2N,$$

where $c_{i,t}={\left[-\sin\left(\phi_{i,t}\right),\cos\left(\phi_{i,t}\right)\right]}^{T}$.

Assuming that the noise term is sufficiently small and introducing weights, $w_{t}=\left[\sqrt{w_{i,t}}\right]$, where $w_{i,t}=P_{i,t}/\sum_{j=1}^{N}P_{j,t}$, in (11) and (12), such that more importance is given to nearby links, gives:

(13a) $$w_{i,t}\mu_{i,t}u_{i,t}^{T}\left(x_{t}-a_{i,t}\right)\approx w_{i,t}\rho ,\;\;\mathrm{for}\;\;i=1,\ldots ,N,$$

(13b) $$w_{i,t}c_{i,t}^{T}\left(x_{t}-a_{i,t}\right)\approx 0,\;\;\mathrm{for}\;\;i=N+1,\ldots ,2N.$$

Therefore, we can rewrite (13) in a linear vector form as:

(14) $$A_{t}y_{t}\approx b_{t},$$

where $y_{t}={\left[x_{t}^{T},\rho \right]}^{T}$, and:

$$A_{t}=\left[\begin{array}{cc} \vdots & \vdots \\ w_{i,t}\mu_{i,t}u_{i,t}^{T} & -w_{i,t}\\ \vdots & \vdots \\ w_{i,t}c_{i}^{T} & 0\\ \vdots & \vdots \end{array}\right]\;,\;b_{t}=\left[\begin{array}{c} \vdots \\ w_{i,t}\mu_{i,t}u_{i,t}^{T}a_{i,t}\\ \vdots \\ w_{i,t}c_{i,t}^{T}a_{i,t}\\ \vdots \end{array}\right]\;.$$

Note that (14) is a linear approximation of (4) for small noise power. The relationship between the two models is described in detail in the text above, and it can be summarized as $z_{t}=b$, $h\left(y_{t}\right)=A_{t}y_{t}$.

By applying the LS criterion to the linearized measurement model in (14), we can approximate the likelihood function in (6) to get an estimate of the target location by solving:

(15) $${\hat{y}}_{t}=\underset{y_{t}={\left[x_{t}^{T},\rho \right]}^{T}}{\arg\;\min}\;\;{∥A_{t}y_{t}-b_{t}∥}^{2},$$

whose solution is readily obtained as ${\hat{y}}_{t}={\left(A_{t}^{T}A_{t}\right)}^{-1}\left(A_{t}^{T}b_{t}\right)$.

Subsequently, one could take advantage of the solution, ${\hat{x}}_{t}={\hat{y}}_{t,1:2}$, where ${\hat{y}}_{t,1:2}$ represents the first two coordinates of the vector ${\hat{y}}_{t}$, obtained through (15), to additionally improve its quality (in the case where the true value of the transmit power is available beforehand, one would simply substitute ${\hat{P}}_{0}$ by $P_{0}$ in the upcoming steps), i.e., the solution could be exploited to find the maximum likelihood (ML) estimate (Note that an estimate of $P_{0}$ could also be obtained directly from the estimate of ρ after solving (15). Nevertheless, our simulations showed that a more precise estimate of $P_{0}$ is obtained through its ML estimation.) of $P_{0}$, ${\hat{P}}_{0}$, as:

(16) $${\hat{P}}_{0}=\frac{\sum_{i=1}^{N}P_{i,t}+10\gamma \log_{10}\frac{∥{\hat{x}}_{t}-a_{i,t}∥}{d_{0}}}{N}.$$

Then, by using $\hat{\rho }=\exp\left(\frac{{\hat{P}}_{0}}{\eta \gamma }\right)$ and following similar steps as described above (note that in this case, we have that the function $h(y_{t})$ depends on the target location only, i.e., $h\left(y_{t}\right)=h\left(x_{t}\right)$), we can rewrite (11) and (12) in a vector form as:

(17) $${\tilde{A}}_{t}x_{t}\approx {\tilde{b}}_{t},$$

where:

$${\tilde{A}}_{t}=\left[\begin{array}{c} \vdots \\ w_{i,t}\mu_{i,t}u_{i,t}^{T}\\ \vdots \\ w_{i,t}c_{i}^{T}\\ \vdots \end{array}\right]\;,\;{\tilde{b}}_{t}=\left[\begin{array}{c} \vdots \\ w_{i,t}\left(\mu_{i,t}u_{i,t}^{T}a_{i,t}+\hat{\rho }\right)\\ \vdots \\ w_{i,t}c_{i,t}^{T}a_{i,t}\\ \vdots \end{array}\right]\;.$$

The model in (17) represents a linear approximation of the measurement model in (4) for known (estimated) target transmit power. Similarly to above, the classical localization problem (disregarding the prior knowledge) can be posed in an LS form:

(18) $${\hat{x}}_{t}=\underset{x_{t}}{\arg\;\min}\;\;{∥{\tilde{A}}_{t}x_{t}-{\tilde{b}}_{t}∥}^{2},$$

whose solution is obtained as ${\hat{x}}_{t}={\left({\tilde{A}}_{t}^{T}{\tilde{A}}_{t}\right)}^{-1}\left({\tilde{A}}_{t}^{T}{\tilde{b}}_{t}\right)$.

## 4. Target Tracking

In this section, we propose two novel tracking algorithms. For both of them, we use the derived linearized measurement model as a base, and we built the new algorithms on top of it. Since in t=0, no prior knowledge is available, we initialize our algorithms by solving (15) and by estimating the transmit power, after which we incorporate the prior knowledge as explained in the remainder of this section. Note that $h\left(y_{t}\right)=h\left(x_{t}\right)$ in the following text, since we estimate the transmit power in t=0. In addition to the tracking algorithms, the proposed sensor navigation technique is described in detail in Section 4.3.

### 4.1. Maximum A Posteriori Estimator

Within the Bayesian methodology, one of the most common criteria for determining a state estimate is the maximum a posteriori (MAP) criterion [19]. According to this estimation approach, we choose a state estimate, ${\hat{\theta }}_{t|t}$, that maximizes the marginal PDF, i.e.,

(19) $${\hat{\theta }}_{t|t}=\underset{\theta_{t}}{\arg\;\max}\;\;p\left(\theta_{t}|z_{1:t}\right).$$

Based on (6), we observe that (19) is equivalent to maximization of $p\left(z_{t}|\theta_{t}\right)p\left(\theta_{t}|z_{1:t-1}\right)$. This is evocative of the ML estimator except for the presence of the prior PDF. Consequently, the MAP estimator is:

(20) $$\begin{array}{c} {\hat{\theta }}_{t|t}=\underset{\theta_{t}}{\arg\;\max}\;\;p\left(z_{t}|\theta_{t}\right)p\left(\theta_{t}|z_{1:t-1}\right)\\ =\underset{\theta_{t}}{\arg\;\max}\;\;\left[\ln p\left(z_{t}|\theta_{t}\right)+\ln p\left(\theta_{t}|z_{1:t-1}\right)\right]. \end{array}$$

The problem in (20) is highly non-convex, and its analytical solution cannot be obtained in closed form, in general. As such, some approximations are required in order to obtain ${\hat{\theta }}_{t|t}$.

First, we can approximate $p(\theta_{t-1}|z_{1:t-1})$ as a Gaussian distribution [29], i.e., $p\left(\theta_{t-1}|z_{1:t-1}\right)∼N\left({\hat{\theta }}_{t-1|t-1},{\hat{\Sigma }}_{t-1|t-1}\right)$. Then, according to (5), we get:

(21) $$p\left(\theta_{t}|z_{1:t-1}\right)\;\approx \;\frac{1}{k_{1}}\;\exp\;\left(\;-\frac{1}{2}{\left(\theta_{t}\;-\;{\hat{\theta }}_{t|t-1}\right)}^{T}{\hat{\Sigma }}_{t|t-1}^{-1}\left(\theta_{t}\;-\;{\hat{\theta }}_{t|t-1}\right)\;\right)\;,$$

where $k_{1}$ is a constant and ${\hat{\theta }}_{t|t-1}$ and ${\hat{\Sigma }}_{t|t-1}$ are the mean and the covariance of the one-step predicted state acquired through (3) as:

(22a) $${\hat{\theta }}_{t|t-1}=S\;{\hat{\theta }}_{t-1|t-1}$$

(22b) $${\hat{\Sigma }}_{t|t-1}=S\;{\hat{\Sigma }}_{t-1|t-1}\;S^{T}+Q.$$

The likelihood function can be written as:

(23) $$p\left(z_{t}|\theta_{t}\right)\;=\;\frac{1}{k_{2}}\exp\;\left(\;-\frac{1}{2}{\left(z_{t}-h\left(x_{t}\right)\right)}^{T}C^{-1}\left(z_{t}-h\left(x_{t}\right)\right)\;\right)\;,$$

where $k_{2}$ is a constant. Then, according to (20), we have:

(24) $$\begin{array}{c} {\hat{\theta }}_{t|t}=\underset{\theta_{t}}{\arg\;\min}\;\;{\left(z_{t}-h\left(x_{t}\right)\right)}^{T}C^{-1}\left(z_{t}-h\left(x_{t}\right)\right)\\ +{\left(\theta_{t}-{\hat{\theta }}_{t|t-1}\right)}^{T}{\hat{\Sigma }}_{t|t-1}^{-1}\left(\theta_{t}-{\hat{\theta }}_{t|t-1}\right). \end{array}$$

We have shown in Section 3 how to approximate the likelihood function by another one, (18), whose closed-form solution is readily available. By following similar reasoning, we can rewrite (24) as:

(25) $${\hat{\theta }}_{t|t}=\underset{\theta_{t}}{\arg\;\min}\;\;{∥H_{t}\theta_{t}-f_{t}∥}^{2},$$

where $H_{t}=\left[{\tilde{A}}_{t},\;0_{2N\times 2};\;{\hat{\Sigma }}_{t|t-1}^{-1/2}\right]$ ($H_{t}\in R^{(2N+4)\times 4}$), where the semi-colon is used to denote a new row entry, $f_{t}=\left[{\tilde{b}}_{t};\;{\hat{\Sigma }}_{t|t-1}^{-1/2}\;{\hat{\theta }}_{t|t-1}\right]$ ($f_{t}\in R^{2N+4}$), and $0_{D\times L}$ is a *D* by *L* matrix of all zeros. The solution of (25) is obtained as ${\hat{\theta }}_{t|t}={\left(H_{t}^{T}H_{t}\right)}^{-1}\left(H_{t}^{T}f_{t}\right)$.

**Figure 1:**
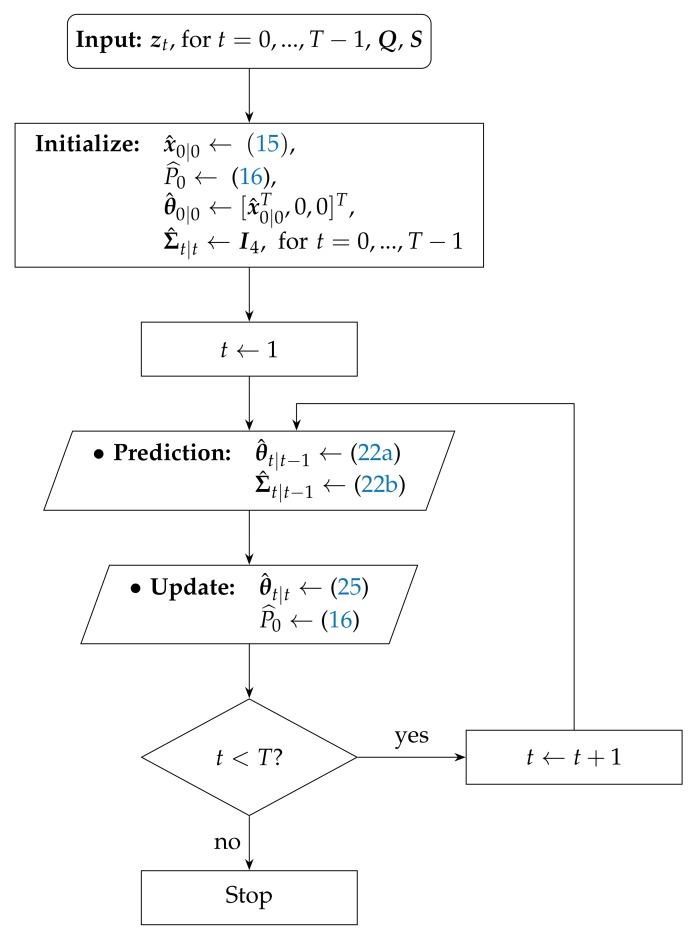
Flowchart of the proposed uMAP algorithm.

The step by step proposed MAP-based algorithm (Notice that in Algorithm 1, we do not update the state covariance matrix. This update could be accomplished through ${\hat{\theta }}_{t|t}$ and the use of Karush–Kuhn–Tucker optimality conditions together with certain approximations (e.g., see the approach in [29]), but we opted not to apply it here. Even though updating the state covariance matrix does bring some gain to our uMAP algorithm, the gain is only marginal, and it does not justify its cost in terms of computational complexity.) for the case where the target transmit power is not known (Note that we do not include $P_{0}$ in the state vector. The reason is that we assume that the transmit power does not change during the entire trajectory. This is a reasonable assumption in practice, since we are tracking the same device during a relatively short period of time.) (labeled here as “uMAP”) is outlined in Algorithm 1, and its flowchart is represented in Figure 1.

**Algorithm 1** uMAP algorithm description.**Require:**
$z_{t}$, for t=0,…,T−1, Q, S 1: **Initialization:**
${\hat{x}}_{0|0}\leftarrow \;\left(15\right)$, ${\hat{P}}_{0}\leftarrow \;\left(16\right)$, ${\hat{\theta }}_{0|0}\leftarrow {\left[{\hat{x}}_{0|0}^{T},0,0\right]}^{T}$, ${\hat{\Sigma }}_{t|t}\leftarrow I_{4}$ for t=0,…,T−1 2: **for**
t=1,…,T−1
**do**
 3:  **Prediction:** 4:    $\begin{array}{c} •\;\;{\hat{\theta }}_{t|t-1}\leftarrow \left(22a\right)\\ •\;\;{\hat{\Sigma }}_{t|t-1}\leftarrow \left(22b\right) \end{array}$ 5:  **Update:** 6:    $\begin{array}{c} •\;\;{\hat{\theta }}_{t|t}\leftarrow \left(25\right)\\ •\;\;{\hat{P}}_{0}\leftarrow \;\left(16\right) \end{array}$ 7: **end for**


### 4.2. Kalman Filter

The Kalman filter (KF) may be thought of as a generalized sequential minimum mean square estimator of a signal embedded in noise, where the unknown parameters are allowed to evolve in time according to a given dynamical model [19]. If the state and the measurement models are linear and the noise is assumed to be zero-mean with finite covariance, the KF provides the optimal solution in the LS sense [2].

Even though the measurement model (4) is non-linear, we can linearize it as in (17). Therefore, by following the KF recipe [19], the mean and the covariance are updated as:

(26a) $${\hat{\theta }}_{t|t}={\hat{\theta }}_{t|t-1}+K_{t}\left({\tilde{b}}_{t}-G_{t}{\hat{\theta }}_{t|t-1}\right),$$

(26b) $${\hat{\Sigma }}_{t|t}=\left(I_{4}-K_{t}G_{t}\right){\hat{\Sigma }}_{t|t-1},$$

where $K_{t}$ is the Kalman gain at time instant *t* and $G_{t}=\left[{\tilde{A}}_{t},0_{2N\times 2}\right]$ ($G_{t}\in R^{2N\times 4}$).

**Figure 2:**
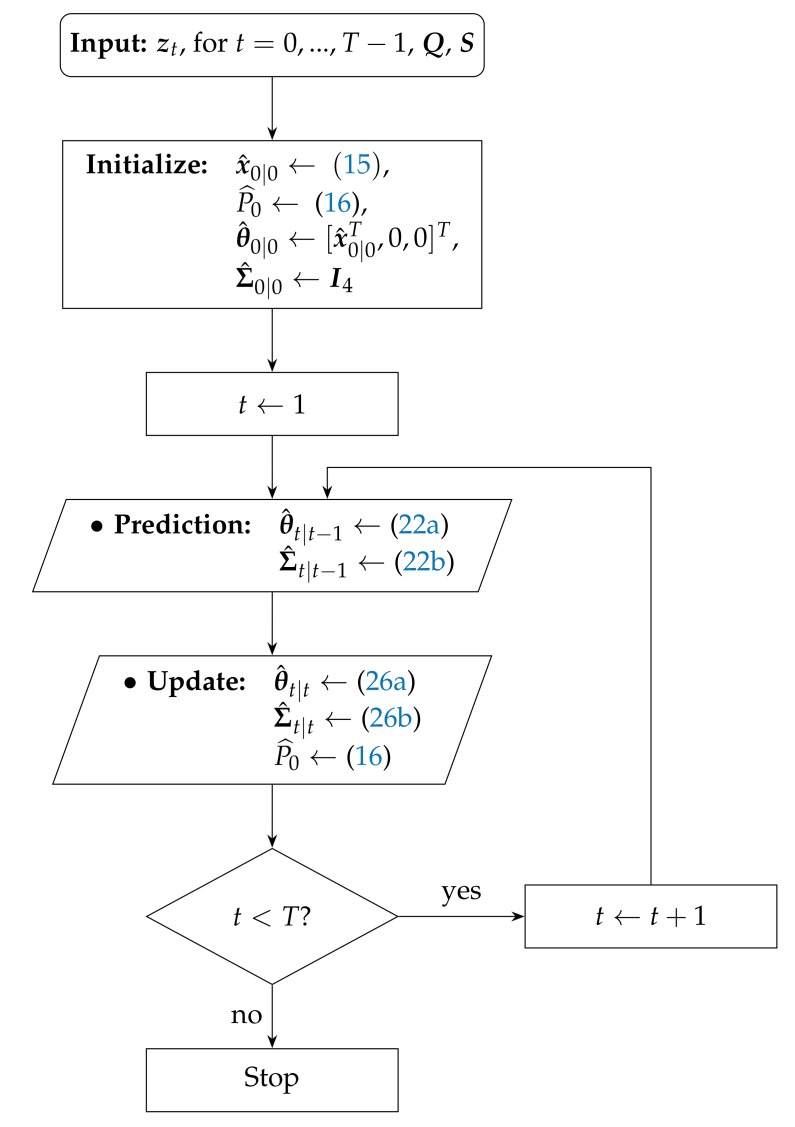
Flowchart of the proposed uKF algorithm.

The step by step proposed KF algorithm (It is worth noting that the KF estimator could also be derived directly from (24) by simply setting the derivative of (24) to zero and then calculating the updated covariance [19] as: ${\hat{P}}_{t|t}=E\left[{\hat{\theta }}_{t|t}{\hat{\theta }}_{t|t}^{T}\right]$, at each time step *t*. However, to do so, one would have to assume the measurement noise covariance to be perfectly known. Since this assumption might not hold in practice, and the measurement model is linearized, a different estimator whose closed-form solution is readily available (25) was derived instead.) for the case where the target transmit power is not known (denoted here as “uKF”) is outlined in Algorithm 2, and its flowchart is represented in Figure 2.

**Algorithm 2** uKF algorithm description.**Require:**
$z_{t}$, for t=0,…,T−1, Q, C, S 1: **Initialization:**
${\hat{x}}_{0|0}\leftarrow \;\left(15\right)$, ${\hat{P}}_{0}\leftarrow \;\left(16\right)$, ${\hat{\theta }}_{0|0}\leftarrow {\left[{\hat{x}}_{0|0}^{T},0,0\right]}^{T}$, ${\hat{\Sigma }}_{0|0}\leftarrow I_{4}$ 2: **for**
t=1,…,T−1
**do**
 3:  **Prediction:** 4:    $\begin{array}{c} •\;\;{\hat{\theta }}_{t|t-1}\leftarrow \left(22a\right)\\ •\;\;{\hat{\Sigma }}_{t|t-1}\leftarrow \left(22b\right) \end{array}$ 5:  $K_{t}\leftarrow {\hat{\Sigma }}_{t|t-1}G_{t}^{T}{\left(G_{t}{\hat{\Sigma }}_{t|t-1}G_{t}^{T}+C\right)}^{-1}$ 6:  **Update:** 7:    $\begin{array}{l} •\;\;{\hat{\theta }}_{t|t}\leftarrow \left(26a\right)\\ •\;\;{\hat{\Sigma }}_{t|t}\leftarrow \left(26b\right)\\ •\;\;{\hat{P}}_{0}\leftarrow \left(16\right) \end{array}$ 8: **end for**

### 4.3. Sensor Navigation

Although the proposed algorithms described in Algorithms 1 and 2 provide efficient solutions to the target tracking problem, their estimation accuracy can be further enhanced. Until now, we have considered the sensors to be static and only the target to be mobile. By allowing sensor mobility, such that they are permitted to move in certain directions based on pre-established rules, we can not only improve the estimation accuracy of the proposed algorithms, but do so with a reduced number of sensors. The price to pay for applying such a routine is somewhat increased computational cost (required for determining the direction of sensor movement) and increased energy consumption (depleted in the process of the actual sensor movement), in comparison with the static sensor routine. Nevertheless, the interest in the target tracking problem using navigated mobile sensors is growing rapidly, especially in areas such as autonomous surveillance, automated data collection and monitoring, to name a few [27,28,33,34].

The proposed routine for sensor navigation is described in Algorithm 3. It represents a universal addition to the proposed uMAP and uKF algorithms, which is realized by incorporating Lines 3–13 after Line 6 in the uMAP and after Line 7 in the uKF. The basic idea of our navigation routine is to let the mobile sensors approach the target with the shortest possible path determined by the available information (such as their estimated and the target’s estimated location) at time instant *t*, until a certain threshold distance, τ. After the mobile sensors penetrate τ, our idea is to spread them around the target, so that we prevent possible sensor collision and possibly loss of information (in case the sensors get physically too close, the acquired measurements could be very similar to one another). More specifically, at Line 4, each mobile sensor estimates its candidate location, ${\overset{˘}{a}}_{i,t}$, by resorting only to its previous estimated location, the already available AoA measurement and its velocity, $v_{a}$. With this candidate location, the mobile sensors then estimate the possible distance from the estimated target location (as if they moved to the candidate location). If this estimated distance is greater than or equal to τ, the candidate location is accepted as the new estimated location of the mobile sensor, ${\hat{a}}_{i,t}$, Line 6; otherwise, the mobile sensors are spread around the target. To this end, we modify the angle measurement, ${\overset{˘}{\phi }}_{i,t-1}$ at Line 9 and use this modified value to estimate the updated location of the mobile sensors, Line 10. However, to account for realistic model mismatches, at Lines 7 and 11, we include noise perturbations within the sensors’ actual movements, which result in imperfect knowledge about the mobile sensors’ locations.

**Algorithm 3** Sensor navigation algorithm description.**Require:**
$a_{i,0}$, $\phi_{i,t}$, for i=1,…,N, t=0,…,T−1, $∥v_{a}∥$, τ  1: **Initialization:**
${\hat{a}}_{i,0}\leftarrow a_{i,0}$  2: **for**
t=1,…,T−1
**do**
  3:  **for**
i=1,…,N
**do**
  4:   
${\overset{˘}{a}}_{i,t}\leftarrow {\hat{a}}_{i,t-1}+∥v_{a}∥\Delta {\left[\cos\left(\phi_{i,t-1}\right),\;\sin\left(\phi_{i,t-1}\right)\right]}^{T}$  5:   **if**
$∥{\overset{˘}{a}}_{i,t}-{\hat{x}}_{t}∥\geq \tau$
**then**
  6:    ${\hat{a}}_{i,t}\leftarrow {\overset{˘}{a}}_{i,t}$  7:    $a_{i,t}\leftarrow a_{i,t-1}+∥v_{a}∥\Delta {\left[\cos\left(\phi_{i,t-1}\right),\;\sin\left(\phi_{i,t-1}\right)\right]}^{T}+r_{x,t}$  8:   **else**  9:    ${\overset{˘}{\phi }}_{i,t}\leftarrow \phi_{i,t}+{\left(-1\right)}^{i}\;\pi /4$ 10:    ${\hat{a}}_{i,t}\leftarrow {\hat{a}}_{i,t-1}+∥v_{a}∥\Delta {\left[\cos\left({\overset{˘}{\phi }}_{i,t-1}\right),\;\sin\left({\overset{˘}{\phi }}_{i,t-1}\right)\right]}^{T}$ 11:    $a_{i,t}\leftarrow a_{i,t-1}+∥v_{a}∥\Delta {\left[\cos\left({\overset{˘}{\phi }}_{i,t-1}\right),\;\sin\left({\overset{˘}{\phi }}_{i,t-1}\right)\right]}^{T}+r_{x,t}$ 12:   **end if** 13:  **end for** 14: **end for**

## 5. Performance Results

**Figure 3:**
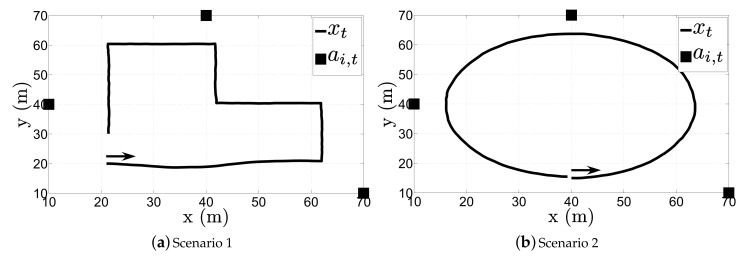
True target trajectory and sensors’ initial locations. The direction of the movement of the target is indicated by the arrow.

In this section, we validate the performance of the proposed algorithms through computer simulations. All of the presented algorithms were solved by using MATLAB. We consider two essentially different scenarios: one in which the target makes sharp maneuvers and another one in which the target constantly changes its direction without making keen maneuvers; see Figure 3. The target state changes according to the state transition model (3), and at each time instant, the radio measurements are generated according to (4). The main simulation parameters are summarized in Table 1. Note that the true value of the PLE for each link was drawn from a uniform distribution on the interval [2.7,3.3], i.e., $\gamma_{i,t}∼U\left[2.7,3.3\right]$, at every time instant. This was done in order to account for a more realistic measurement model mismatch. Moreover, in the case where sensor mobility is allowed, we set a threshold distance (In our simulations, we have also studied the influence of this parameter on the performance of the proposed algorithms. It was concluded that it has no significant impact on the performance, since similar results were attained for different values of τ (e.g., τ=10 m or τ=0.5 m). However, we chose this particular value because it seems a reasonable practical threshold, keeping in mind the estimation error and noise influence to prevent sensor collision.) so that we avoid physical collisions between the sensors. The performance metric used here is the root mean square error (RMSE), defined as ${\mathrm{RMSE}}_{t}=\sqrt{\sum_{i=1}^{M_{c}}\frac{∥x_{i,t}-{\hat{x}}_{i,t}{∥}^{2}}{M_{c}}},$ where ${\hat{x}}_{i,t}$ denotes the estimate of the true target location, $x_{i,t}$, in the *i*-th $M_{c}$ run at time instant *t*.

The performance of the proposed uMAP and uKF algorithms is compared with the existing KF in [6], where the initial target state (Although this initialization seems fair, in our simulations, we also considered initializing the KF in [6] in the same way as the proposed algorithms. Even for such an initialization, the proposed algorithms still outperformed the KF in [6].) was obtained by solving the LS method used in [6] to linearize the observation model (since the authors in [6] proposed also a method for PLE estimation, in order to make the comparison fair, the true value of the PLE for every link is considered perfectly known for the KF [6] at any time step). Furthermore, we compare the performance of the proposed algorithms with some well-known and more traditional methods that were not specifically designed for RSS-AoA target tracking, but whose generalization to this scenario is straightforward, i.e., we compare it with the performance of the EKF [19,20,21,22], UKF [23,24,25] and PF [25,26] algorithms, labeled accordingly. To make the comparison as fair as possible, these algorithms are initialized in the same manner as the proposed algorithms (In our simulations, we also examined the influence of the initialization on these algorithms and on the proposed ones. We considered a random initialization, as well as the initialization in the center of the considered area. It is worth mentioning that the proposed algorithms always converged, independent of the initialization used, while the EKF, UKF and PF did not always converge.). Moreover, in favor of testing the belief that the Bayesian approaches (which integrate the prior knowledge with observations) outperform the classical ones (which disregard the prior knowledge and are based merely on observations), we show the results for the sequential localization method in (18) with perfect knowledge about the target transmit power and PLE, denoted here by “WLS”. Finally, to offer a lower bound on the performance of the proposed algorithms their counterparts for known target transmit power are also included, labeled here as “MAP” and “KF”.

**Figure 4:**
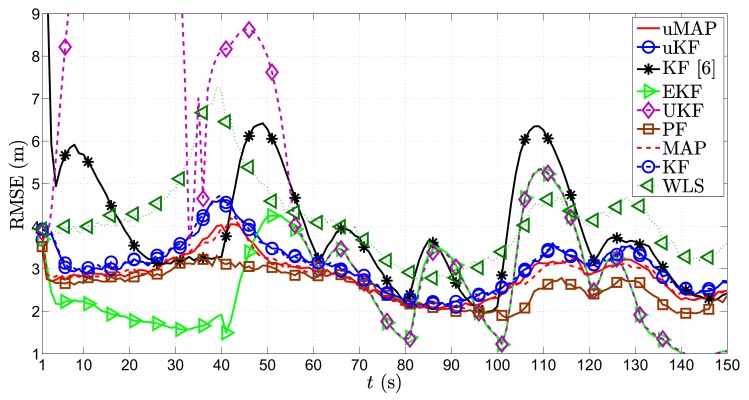
RMSE (m) versus *t* (s) comparison in the first scenario, when N=3, $∥v_{a}∥=0$ m/s, $\sigma_{n_{i}}=9$ dB, $\sigma_{m_{i}}=4\frac{\pi }{180}$ rad, γ=3, $\gamma_{i}∼U\left[2.7,3.3\right]$, $P_{0}=-10$ dBm, $q=2.5\times 10^{-3}\;m^{2}/s^{3}$, $M_{c}=1000$.

Figure 4 illustrates the RMSE (m) versus *t* (s) comparison of all considered approaches in the first scenario, for the static sensors case, i.e., $∥v_{a}∥=0$ m/s. From it, we can observe that all algorithms suffer deteriorations at each sharp maneuver of the target, especially in the proximity of the sensors. This is somewhat anticipated, since the role of the prior knowledge is canceled out with each sharp maneuver, and the vicinity of the target and any of the sensors creates an unbalance between the significance of that particular measurement and all of the other ones. Nonetheless, all algorithms recover fairly quickly from these impairments. Furthermore, the figure shows that the proposed algorithms outperform the existing KF in [6] in general, as well as the naive WLS approach that only makes use of the observations and disregards the prior knowledge for all *t*. Moreover, it can be seen that the PF algorithm shows the most stable performance throughout the whole target trajectory and that it slightly outperforms the proposed algorithms. Furthermore, it is worth mentioning that our algorithms show robustness to not knowing the target transmit power, since they achieve their lower bounds given by their equivalents for known transmit power. Finally, the new algorithms behave excellent performance even for the case where the PLE is not perfectly known.

**Figure 5:**
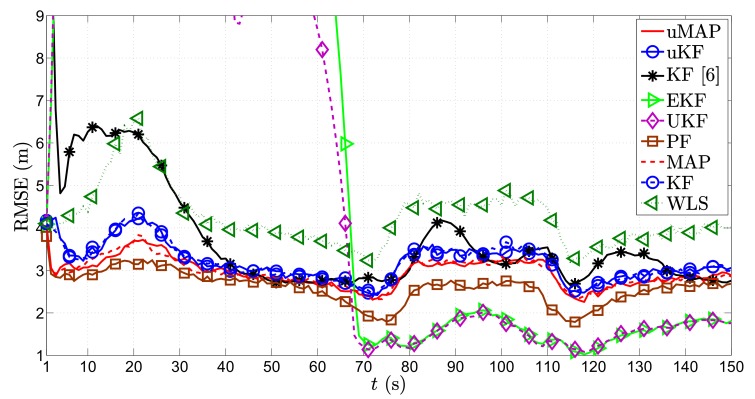
RMSE (m) versus *t* (s) comparison in the second scenario, when N=3, $∥v_{a}∥=0$ m/s, $\sigma_{n_{i}}=9$ dB, $\sigma_{m_{i}}=4\frac{\pi }{180}$ rad, γ=3, $\gamma_{i}∼U\left[2.7,3.3\right]$, $P_{0}=-10$ dBm, $q=2.5\times 10^{-3}\;m^{2}/s^{3}$, $M_{c}=1000$.

Figure 5 illustrates the RMSE (m) versus *t* (s) comparison of all considered approaches in the second scenario, for the static sensor case. From the figure, one can observe that the performance of most considered algorithms is significantly smoother in comparison with the first scenario. This behavior is not surprising, since the target, although constantly changing its direction, is moving much more smoothly now. Nevertheless, the figure shows that the EKF and UKF experience significant performance deterioration during the initial target trajectory. Even though their performance recovers in the later phase of the target trajectory, their overall localization accuracy is poor in the considered scenario. This can be explained to some extent by the fact that, in the case of EKF, the error propagation is not approximated sufficiently well by first-order Taylor series approximations, while for the case of UKF, one has to choose values of certain parameters (e.g., the parameter κ in [23], which scales the sigma points of the unscented transformation towards or away from the mean of the prior distribution), which is usually done in a heuristic manner and might affect the performance of the UKF in some scenarios (This problem of convergence for EKF and UKF might be solved by, e.g., introducing additional constraints in the two sequential estimates of the algorithms such that large leaps in the estimation process are avoided, but this would increase their computational complexity and could also affect their overall performance. Furthermore, in the case of the EKF, applying second-order Taylor series expansion to approximate the original system could resolve this problem [21], but this comes at the cost of increased computational complexity (computation of the Hessian matrix)). Furthermore, Figure 5 shows that the proposed algorithms match the performance of the PF and outperform the existing KF in general, and they show robustness to uncertainty in the transmit power.

We present the average RMSE, $\bar{\mathrm{RMSE}}$ (m), performance of the considered algorithms for static sensors in both scenarios in Table 2. From the table, we can see that the proposed uMAP algorithm performs very close to the PF in both scenarios and that the proposed linearization technique offers an improvement of roughly 1 m in both scenarios, in comparison with the existing one [6].

In terms of computational complexity, the most expensive operation of an algorithm is matrix multiplication and inversion [35]. This operation is a part of practically all considered algorithms (except for PF, but this algorithm has other drawbacks related to the number of particles used). Nevertheless, the UKF and the PF use a number of sigma points and particles, respectively, which affect their computational complexities and consequently their execution times, as can be seen from Table 3.

**Figure 6:**
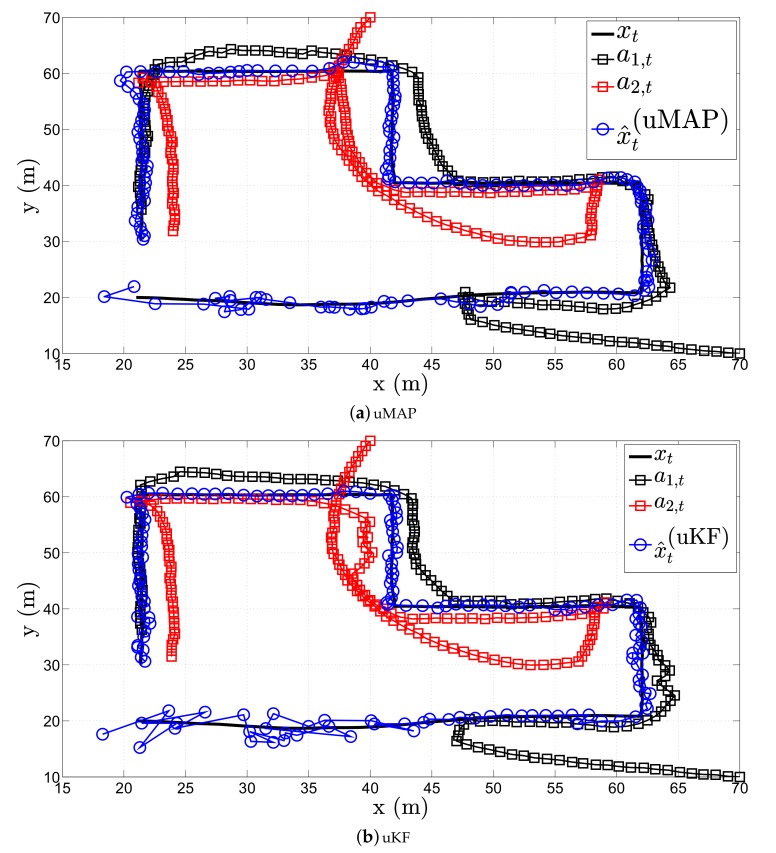
Illustration of the estimation process in the first scenario, when N=2, $∥v_{a}∥=1$ m/s, $\sigma_{n_{i}}=9$ dB, $\sigma_{m_{i}}=4\frac{\pi }{180}$ rad, γ=3, $\gamma_{i}∼U\left[2.7,3.3\right]$, τ=5 m, $P_{0}=-10$ dBm, $q=2.5\times 10^{-3}\;m^{2}/s^{3}$.

Figure 6 illustrates a realization of the estimation process in the first scenario of the proposed (a) uMAP and (b) uKF algorithm, respectively, when sensor mobility is allowed. Hereafter, we only use N=2 mobile sensors, and in particular, we use the first two sensors from the Table 1. From the figure, one can observe that both proposed algorithms solve very efficiently the target tracking problem with only N=2 sensors (each sensors measures both RSS and AoA values), owing to their mobility. However, it can be seen that both uMAP and uKF algorithms require a number of iterations to stabilize their performance. Nonetheless, this behavior is expected since we only use N=2 sensors in this case.

**Figure 7:**
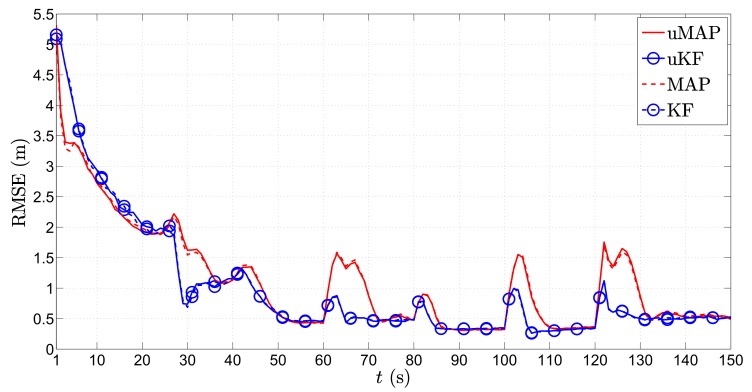
RMSE (m) versus *t* (s) comparison in the first scenario, when N=2, $∥v_{a}∥=1$ m/s, $\sigma_{n_{i}}=9$ dB, $\sigma_{m_{i}}=4\frac{\pi }{180}$ rad, γ=3, $\gamma_{i}∼U\left[2.7,3.3\right]$, τ=5 m, $P_{0}=-10$ dBm, $q=2.5\times 10^{-3}\;m^{2}/s^{3}$, $M_{c}=1000$.

Figure 7 depicts the RMSE (m) versus *t* (s) performance comparison of the proposed algorithms in the first scenario for the mobile sensor case. As foreseen, the figure shows the poorest estimation accuracy in the first few time steps, which generally improves with time. This is because, in the first few time instants, the mobile sensors are far away from the target, and as they get closer to it, the performance improves in general. Essentially, impairments occur only at the critical points when the target makes sharp turns. However, even though we use only N=2 sensors now, due to their mobility, we can see that these deteriorations are notably milder in comparison with the static N=3 sensors case (Figure 4). Moreover, the proposed uKF algorithm slightly outperforms the proposed uMAP. Lastly, the new algorithms show exceptional behavior even for the case where the PLE and the true mobile sensors’ locations are not perfectly known.

**Figure 8:**
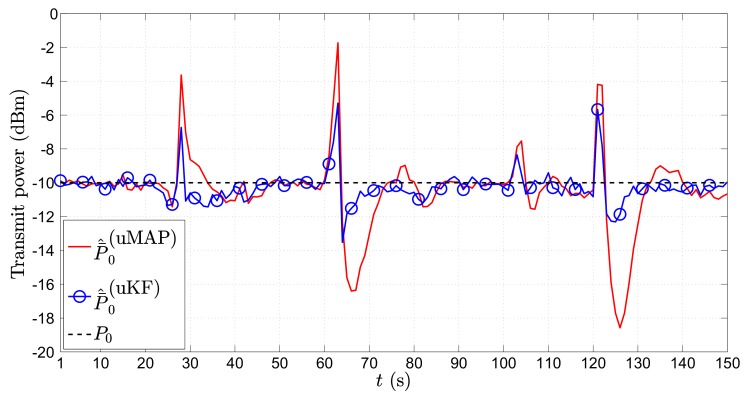
${\hat{\bar{P}}}_{0}$ (dBm) versus *t* (s) comparison in the first scenario, when N=2, $∥v_{a}∥=0$ m/s, $\sigma_{n_{i}}=9$ dB, $\sigma_{m_{i}}=4\frac{\pi }{180}$ rad, γ=3, $\gamma_{i}∼U\left[2.7,3.3\right]$, τ=5 m, $P_{0}=-10$ dBm, $q=2.5\times 10^{-3}m^{2}/s^{3}$, $M_{c}=1000$.

It might also be of interest for some applications to get an estimate of the target’s transmit power. Hence, in Figure 8, we show the average ML estimate of $P_{0}$, ${\hat{\bar{P}}}_{0}$ (dBm), in the first scenario through time *t* (s) for the mobile sensors case. From Figure 8, we can see that both proposed algorithms provide an excellent estimate of the transmit power in general. Similar to the case of location estimation, the only significant impairments in the power estimates occur at the critical points.

**Figure 9:**
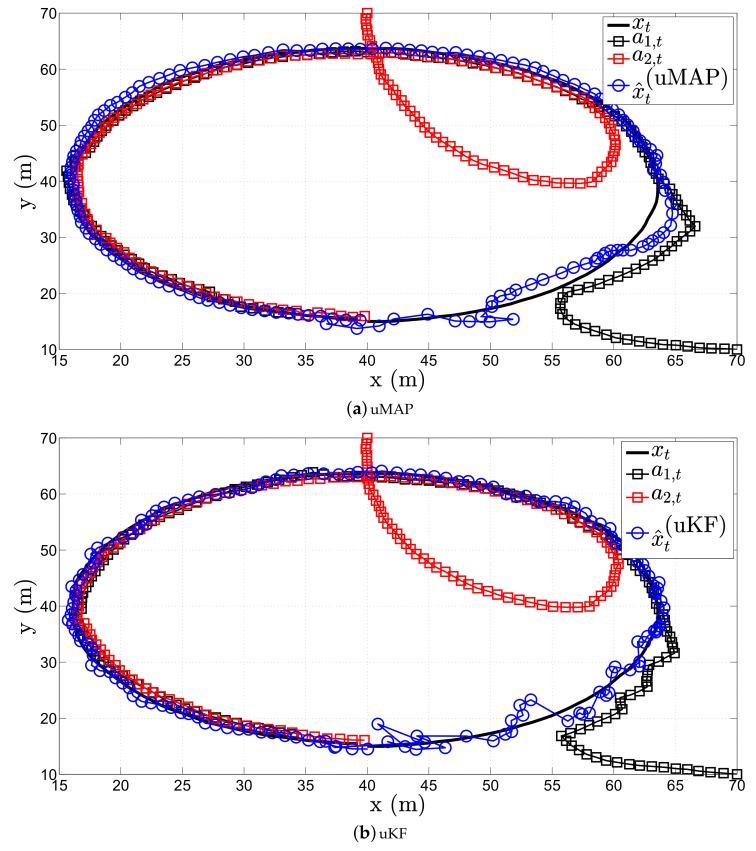
Illustration of the estimation process in the second scenario, when N=2, $∥v_{a}∥=1$ m/s, $\sigma_{n_{i}}=9$ dB, $\sigma_{m_{i}}=4\frac{\pi }{180}$ rad, γ=3, $\gamma_{i}∼U\left[2.7,3.3\right]$, τ=5 m, $P_{0}=-10$ dBm, $q=2.5\times 10^{-3}\;m^{2}/s^{3}$.

Figure 9 illustrates a realization of the estimation process in the second scenario of the proposed (a) uMAP and (b) uKF algorithms, respectively, when sensor mobility is allowed. As in the first scenario, both proposed algorithms show exceptionally good performance.

**Figure 10:**
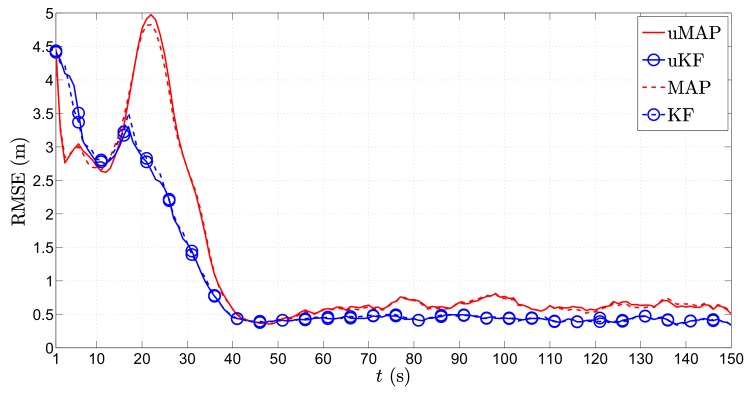
RMSE (m) versus *t* (s) comparison in the second scenario, when N=2, $∥v_{a}∥=1$ m/s, $\sigma_{n_{i}}=9$ dB, $\sigma_{m_{i}}=4\frac{\pi }{180}$ rad, γ=3, $\gamma_{i}∼U\left[2.7,3.3\right]$, τ=5 m, $P_{0}=-10$ dBm, $q=2.5\times 10^{-3}\;m^{2}/s^{3}$, $M_{c}=1000$.

Figure 10 depicts the RMSE (m) versus *t* (s) performance comparison of the proposed algorithms in the second scenario for the mobile sensors case. The figure reveals that both proposed algorithm require a certain amount of time before they catch up with the target, after which their estimation performance is outstanding and quite stable. Furthermore, a somewhat better performance of the proposed uKF can be seen in comparison with the uMAP.

**Figure 11:**
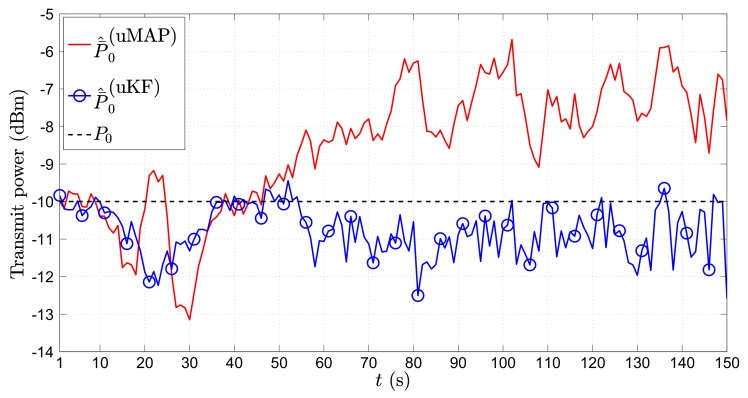
${\hat{\bar{P}}}_{0}$ (dBm) versus *t* (s) comparison in the second scenario, when N=2, $∥v_{a}∥=0$ m/s, $\sigma_{n_{i}}=9$ dB, $\sigma_{m_{i}}=4\frac{\pi }{180}$ rad, γ=3, $\gamma_{i}∼U\left[2.7,3.3\right]$, τ=5 m, $P_{0}=-10$ dBm, $q=2.5\times 10^{-3}\;m^{2}/s^{3}$, $M_{c}=1000$.

In Figure 11, we present the ${\hat{\bar{P}}}_{0}$ (dBm) versus *t* (s) performance comparison in the second scenario for the mobile sensors case. Compared with the results in the first scenario, we can see that the estimation accuracy of $P_{0}$ is not as good. This result is interesting on its own, and it seems to be an outcome of the peculiarity of the target’s trajectory (constant change of direction). Nonetheless, a detailed analysis of this phenomenon is beyond the scope of this work. Furthermore, it can be noticed that a considerably better $P_{0}$ estimate is obtained through the proposed uKF.

**Figure 12:**
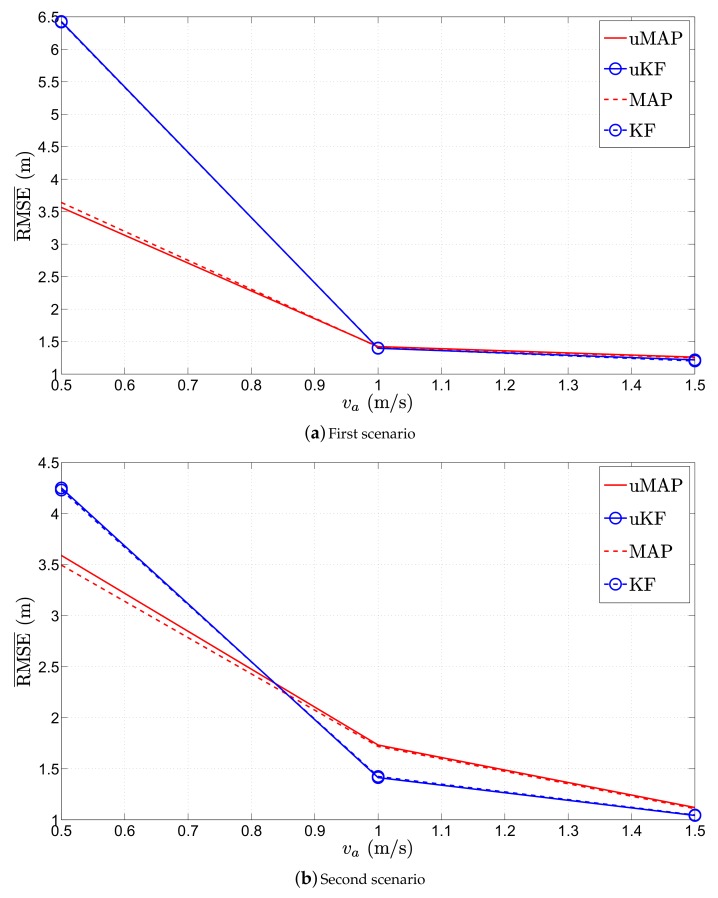
$\bar{\mathrm{RMSE}}$ (m) versus $∥v_{a}∥$ (m/s) comparison, when N=2, $\sigma_{n_{i}}=9$ dB, $\sigma_{m_{i}}=4\frac{\pi }{180}$ rad, $∥v_{t}∥=1$ m/s, γ=3, $\gamma_{i}∼U\left[2.7,3.3\right]$, τ=5 m, $P_{0}=-10$ dBm, $q=2.5\times 10^{-3}\;m^{2}/s^{3}$, $M_{c}=1000$.

It would also be interesting to investigate the influence of the mobile sensor’s velocity on the performance of the proposed algorithms. Consequently, we present the $\bar{\mathrm{RMSE}}$ (m) versus $∥v_{a}∥$ (m/s) performance comparison for the first and the second scenario in Figure 12. From the figure, it is obvious that the performance of the proposed algorithms depends on the sensor’s velocity, and one can notice that the performance of all algorithms improves as $∥v_{a}∥$ (m/s) is increased. This is somewhat intuitive, since the mobile sensors catch the target more rapidly as they move at higher velocity. Moreover, the overall performance of the proposed algorithms is very good, while for $∥v_{a}∥\geq ∥v_{t}∥$, their performance is remarkable.

## 6. Conclusions

In this work, we have addressed the target tracking problem in WSN where sensor mobility was allowed. The mobile sensors made use of uncalibrated RSS measurements, where imperfect knowledge about the PLE and unknown target transmit power was considered, which were combined with AoA observations. We have shown that this highly non-linear measurement model can be linearized by applying the described procedure. Then, by following the Bayesian methodology, we have managed to integrate the prior knowledge (extracted from the state transition model) with the observations in order to further improve the estimation accuracy. As a result of our work, two novel tracking algorithms were proposed, namely uMAP and uKF. Furthermore, a simple navigation procedure was proposed, which even further improved the estimation accuracy of our algorithms. The new algorithms were compared with the existing KF algorithm for RSS-AoA target tracking, as well as some general ones, such as the EKF, UKF and PF. Furthermore, the proposed algorithms were compared with the traditional approach, which neglects the prior knowledge and makes use of observations exclusively. Two different scenarios were considered: where the target makes sharp maneuvers and where the target follows a smoother trajectory. Extensive simulations have been carried out, and the results have confirmed that incorporation of the prior knowledge into an estimator can significantly improve its estimation accuracy. Furthermore, the simulation results showed that the proposed linearization technique offers significant error reduction in comparison with the existing one [6]. Our simulations have shown also that the proposed algorithms converge in all considered scenarios, while the same cannot be said for the general ones. Moreover, the results corroborated the usefulness of the proposed mobile sensor navigation routine, demonstrating not only a remarkable improvement in the estimation accuracy, but doing so with a reduced number of sensors. Finally, the proposed algorithms exhibited robustness to not knowing the transmit power, as well as to imperfect knowledge about the PLE and the true sensors’ locations.

## Figures and Tables

**Table 1:** Summary of the simulation parameters. PLE, path loss exponent; PF, particle filter.

Parameter	Description	Value
*N*	The number of sensors	3 (for static case) or 2 (for mobile case)
$a_{i,0}$	The true initial sensor locations	$\left[{\left[70,10\right]}^{T},{\left[40,70\right]}^{T}{\left[10,40\right]}^{T}\right]$ (m)
$P_{0}$	The reference power	−10 (dBm)
γ	The assumed PLE	3
$\gamma_{i,t}$	The true PLE for *i*-th link	$\gamma_{i,t}∼U\left[2.7,3.3\right]$
Δ	The sampling interval	1 (s)
*T*	The duration of the trajectory	150 (s)
$M_{c}$	The number of Monte Carlo runs	1000
$∥v_{t}∥$	The target’s speed	1 (m/s)
$∥v_{a}∥$	The sensors’ speed	Varies in the simulations
τ	The threshold distance	5 (m)
$\sigma_{n_{i}}$	The RSS noise power	9 (dB)
$\sigma_{m_{i}}$	The AoA noise power	4 (degrees)
*q*	The state process noise	$2.5\times 10^{-3}$ ($m^{2}/s^{3}$)
$N_{\mathrm{PF}}$	The number of particles in the PF	200

**Table 2:** $\bar{\mathrm{RMSE}}$ (m) of the considered algorithms.

Algorithm	uMAP	uKF	KF [6]	EKF	UKF	PF	MAP	KF	WLS
**Scenario 1**	2.88	3.15	4.31	2.78	7.16	2.60	2.87	3.13	4.22
**Scenario 2**	2.97	3.22	4.14	24	22	2.63	2.97	3.22	4.30

**Table 3:** Running time (s) of the considered algorithms for $M_{c}=1000$. Processor: Intel(R)Core(TM)i7-4710HQ, CPU@2.50 GHz.

Algorithm	uMAP	uKF	KF [6]	EKF	UKF	PF	WLS
**Scenario 1**	36.0	31.3	31.2	37.0	54.1	467.6	28.8
**Scenario 2**	36.1	31.4	31.9	37.1	55.2	468.9	28.8
